# Homeostatic iron regulatory protein drives glioblastoma growth via tumor cell-intrinsic and sex-specific responses

**DOI:** 10.1093/noajnl/vdad154

**Published:** 2023-11-28

**Authors:** Katie M Troike, Sabrina Z Wang, Daniel J Silver, Juyeun Lee, Erin E Mulkearns-Hubert, Nicole Hajdari, Prabar K Ghosh, Kristen E Kay, Julia L Beilis, Sofia E Mitchell, Christopher W Bishop, Ellen S Hong, Mykyta Artomov, Christopher G Hubert, Prajwal Rajappa, James R Connor, Paul L Fox, Bjarne W Kristensen, Justin D Lathia

**Affiliations:** Department of Cardiovascular and Metabolic Sciences, Lerner Research Institute, Cleveland Clinic, Cleveland, Ohio, USA; Department of Molecular Medicine, Lerner College of Medicine, Case Western Reserve University, Cleveland, Ohio, USA; Department of Cardiovascular and Metabolic Sciences, Lerner Research Institute, Cleveland Clinic, Cleveland, Ohio, USA; Medical Scientist Training Program, Case Western Reserve University School of Medicine, Cleveland, Ohio, USA; Department of Pathology, Case Western Reserve University, Cleveland, Ohio, USA; Department of Cardiovascular and Metabolic Sciences, Lerner Research Institute, Cleveland Clinic, Cleveland, Ohio, USA; Case Comprehensive Cancer Center, Case Western Reserve University, Cleveland, Ohio, USA; Department of Cardiovascular and Metabolic Sciences, Lerner Research Institute, Cleveland Clinic, Cleveland, Ohio, USA; Department of Cardiovascular and Metabolic Sciences, Lerner Research Institute, Cleveland Clinic, Cleveland, Ohio, USA; Department of Cardiovascular and Metabolic Sciences, Lerner Research Institute, Cleveland Clinic, Cleveland, Ohio, USA; Department of Cardiovascular and Metabolic Sciences, Lerner Research Institute, Cleveland Clinic, Cleveland, Ohio, USA; Department of Cardiovascular and Metabolic Sciences, Lerner Research Institute, Cleveland Clinic, Cleveland, Ohio, USA; Department of Molecular Medicine, Lerner College of Medicine, Case Western Reserve University, Cleveland, Ohio, USA; Department of Cardiovascular and Metabolic Sciences, Lerner Research Institute, Cleveland Clinic, Cleveland, Ohio, USA; Department of Cardiovascular and Metabolic Sciences, Lerner Research Institute, Cleveland Clinic, Cleveland, Ohio, USA; Department of Cardiovascular and Metabolic Sciences, Lerner Research Institute, Cleveland Clinic, Cleveland, Ohio, USA; Department of Cardiovascular and Metabolic Sciences, Lerner Research Institute, Cleveland Clinic, Cleveland, Ohio, USA; Medical Scientist Training Program, Case Western Reserve University School of Medicine, Cleveland, Ohio, USA; Institute for Genomic Medicine, Nationwide Children’s Hospital, Columbus, Ohio, USA; Department of Pediatrics, The Ohio State Wexner Medical Center, Columbus, Ohio, USA; Department of Cardiovascular and Metabolic Sciences, Lerner Research Institute, Cleveland Clinic, Cleveland, Ohio, USA; Case Comprehensive Cancer Center, Case Western Reserve University, Cleveland, Ohio, USA; Department of Biochemistry, Case Western Reserve University, Cleveland, Ohio, USA; Institute for Genomic Medicine, Nationwide Children’s Hospital, Columbus, Ohio, USA; Department of Neurological Surgery, The Ohio State Wexner Medical Center, Columbus, Ohio, USA; Department of Neurosurgery, Penn State Hershey Medical Center, Hershey, Pennsylvania, USA; Department of Cardiovascular and Metabolic Sciences, Lerner Research Institute, Cleveland Clinic, Cleveland, Ohio, USA; Case Comprehensive Cancer Center, Case Western Reserve University, Cleveland, Ohio, USA; Department of Clinical Medicine, Biotech Research and Innovation Center (BRIC), University of Copenhagen, Copenhagen, Denmark; Department of Cardiovascular and Metabolic Sciences, Lerner Research Institute, Cleveland Clinic, Cleveland, Ohio, USA; Department of Molecular Medicine, Lerner College of Medicine, Case Western Reserve University, Cleveland, Ohio, USA; Case Comprehensive Cancer Center, Case Western Reserve University, Cleveland, Ohio, USA; Rose Ella Burkhardt Brain Tumor and Neuro-Oncology Center, Cleveland Clinic, Cleveland, Ohio, USA

**Keywords:** glioblastoma, HFE, iron, reactive oxygen species, sex difference

## Abstract

**Background:**

Glioblastoma (GBM) displays alterations in iron that drive proliferation and tumor growth. Iron regulation is complex and involves many regulatory mechanisms, including the homeostatic iron regulator (*HFE*) gene, which encodes the homeostatic iron regulatory protein. While *HFE* is upregulated in GBM and correlates with poor survival outcomes, the function of HFE in GBM remains unclear.

**Methods:**

We interrogated the impact of cell-intrinsic *Hfe* expression on proliferation and survival of intracranially implanted animals through genetic gain- and loss-of-function approaches in syngeneic mouse glioma models, along with in vivo immune assessments. We also determined the expression of iron-associated genes and their relationship to survival in GBM using public data sets and used transcriptional profiling to identify differentially expressed pathways in control compared to *Hfe*-knockdown cells.

**Results:**

Overexpression of *Hfe* accelerated GBM proliferation and reduced animal survival, whereas suppression of *Hfe* induced apoptotic cell death and extended survival, which was more pronounced in females and associated with attenuation of natural killer cells and CD8+ T cell activity. Analysis of iron gene signatures in *Hfe*-knockdown cells revealed alterations in the expression of several iron-associated genes, suggesting global disruption of intracellular iron homeostasis. Further analysis of differentially expressed pathways revealed oxidative stress as the top pathway upregulated following *Hfe* loss. *Hfe* knockdown indeed resulted in enhanced ^55^Fe uptake and generation of reactive oxygen species.

**Conclusions:**

These findings reveal an essential function for HFE in GBM cell growth and survival, as well as a sex-specific interaction with the immune response.

**Key Points**

- Homeostatic iron regulator (HFE) is upregulated in glioblastoma and negatively impacts survival.
- *Hfe* knockdown reduced tumor cell growth and increased cell death, leading to increased animal survival in vivo, with opposite effects after *Hfe* overexpression.
- The in vivo response to *Hfe* modulation is more pronounced in female mice and associated with natural killer cells and CD8+ T cell activity.

**Importance of the Study**

Dysregulation of iron metabolism is an important feature of glioblastoma (GBM) that contributes to tumor growth and negatively impacts survival. Iron regulation is a complex process that involves many pathway members, but the individual function of these genes is not well elucidated. We identify homeostatic iron regulator (HFE), a critical regulator of iron homeostasis, as important for GBM cell growth and survival. Our findings also suggest a sex difference in HFE where females are more sensitive to HFE alterations, which impact antitumor natural killer cell and CD8+ T cell activity. This ultimately results in differential survival outcomes in which females show a greater difference in HFE-dependent survival in preclinical model and human GBM patient outcome. Our findings demonstrate that HFE enables tumor cell proliferation and survival in GBM in a sex-specific manner and may be a viable target for modulating tumor iron flux, altering immune response, and inducing apoptosis in tumor cells.

Glioblastoma (GBM) is the most common primary malignant brain tumor and has an exceptionally poor prognosis. When treated with standard-of-care therapy, which includes maximal safe surgical resection, radiation, and chemotherapy, the median survival for patients with GBM is approximately 17–20 months.^1^ Multiple factors have been identified that facilitate GBM growth and therapeutic resistance, culminating in disease recurrence. Tumor cell-intrinsic factors, including cellular heterogeneity, metabolic, and genetic aberrations, along with extrinsic factors within the neural, endothelial, and immune cell compartments, drive tumor progression and contribute to therapeutic failure.^2^ Recently, patient sex has been reported as a determinant of incidence and survival in GBM, with male patients exhibiting greater risk of developing GBM, as well as worse prognosis, when compared to female patients.^3^ Sex differences are mediated through the effects of sex chromosomes and sex hormones, which influence a variety of processes in GBM, including malignant transformation, epigenetic landscape, antitumor immunity, and therapeutic response.^4–7^ As such, the intersection of sex and other tumor cell-intrinsic and cell-extrinsic features is a critical area of investigation. One biological process known to exhibit a large degree of sexual differences and to drive radioresistance and tumor progression in GBM is iron metabolism, an attractive focus for therapeutic development.^8,9^

Iron is an indispensable element required for enzymatic function in a number of normal cellular processes such as ATP production, DNA synthesis, and cell cycle regulation.^10^ The brain is dependent on iron for normal development and function, playing a role in neurotransmitter synthesis, myelination, and microglia polarization.^11^ The redox cycling potential of iron contributes to its protumorigenic effects, including the generation of free radicals, which can damage DNA and promote malignant transformation.^10^ Therefore, a complex regulatory mechanism involving iron uptake, storage, and release, mediated by a variety of iron-handling proteins, ensures the tight maintenance of intracellular iron levels to prevent toxicity. Dysregulation of iron metabolism, primarily driven by the aberrant expression of iron-handling proteins, is a hallmark of the tumor state, and increased import accompanied by reduced export is frequently observed in many different cancers.^12^ Iron is therefore a putative therapeutic target for cancer, although the use of iron chelators in vivo has been limited by their lack of tumor specificity and their side effects.^13^ GBM tumors exhibit increased iron uptake compared to nontumor brain tissue, a property that has been exploited for specific targeting and imaging of these lesions.^14,15^ Previous work has demonstrated that cancer stem-like cells within GBM tumors are efficient iron scavengers, upregulating their expression of specific iron-handling proteins to enhance uptake and storage and drive proliferation.^16^ Conversely, disrupting intracellular iron storage in GBM cells induces cell death by multiple parallel mechanisms, including iron-dependent ferroptosis.^17^ Given the pivotal role of iron in tumor initiation and growth, further insight into its regulation in GBM is necessary to improve understanding of this disease and to aid in the development of new therapies.

In a normal cellular state, iron homeostasis is maintained through a tightly regulated balance of iron import, export, and storage. When this balance is disrupted, iron can accumulate and lead to pathologies such as hereditary hemochromatosis (HH), an iron overload disorder. Dysregulated iron absorption in HH causes tissue iron accumulation, culminating in oxidative damage and cell death.^18^ Additionally, ferroptosis, an iron-dependent form of cell death, has been reported to contribute to tissue damage in mouse models of hemochromatosis.^19^ Most cases of HH are driven by mutations in the homeostatic iron regulator (*HFE*) gene, which encodes the transmembrane HFE protein.^20^ In normal cells, HFE functions as a cellular iron sensor, mediating the uptake and release of iron indirectly through interactions with other iron-associated proteins.^21^ Prior work in GBM has demonstrated an inverse relationship between *HFE* expression and survival in female patients.^22^ However, no study to date has mechanistically clarified how HFE modulates GBM patient survival. Here, we demonstrate that *Hfe* loss in glioma cells enhances iron uptake and the generation of reactive oxygen species (ROS), which in turn promotes tumor cell death and extends survival. In addition, *Hfe* manipulation was subject to sex differences in immune response, which likely underscores the sex differences observed in GBM patient outcomes. Taken together, these findings highlight the importance of HFE in iron homeostatic maintenance and its potential as a target for therapeutic management of GBM.

## Materials and Methods

### Perl’s Prussian Blue Staining

Formalin-fixed paraffin-embedded patient samples were assembled as tissue microarrays and kindly provided by Dr. Bjarne Kristensen with approval by the Regional Committee on Health Research Ethics for Southern Denmark (S-20150148). Staining of tumor sections with Perl’s Prussian blue and nuclear fast red counterstain was performed by the Lerner Research Institute Imaging Core. Images were acquired on a Leica Aperio slide scanner using a 20× objective.

### Patient mRNA Expression and Survival

Clinical and microarray expression data for the *IDH*-wild-type subset of the glioblastoma cohort of TCGA was downloaded from the GlioVis portal (http://gliovis.bioinfo.cnio.es).^23^ Additionally, mutational data were obtained using RTCGAToolbox.^24^ Wild-type *IDH1* status was confirmed by inspecting the somatic mutations in the analyzed cohort. Cox proportional hazard model from the survival R package^25^ was used to evaluate effects of *HFE* expression on overall survival. Previous findings indicated that there is a complex interaction among age, sex, and *HFE* expression.^22^ We therefore updated the survival model to address previous statistical analyses challenges. For the full cohort analysis, we used the following covariates: Diagnosis Age, Sex, O^6^-methylguanine-DNA methyltransferase (MGMT) methylation status, and the interaction terms—Diagnosis Age × Sex, MGMT methylation status × Sex, HFE median expression group × Sex. Interaction terms were used to account for the potential presence of sex-specific effects. Similarly, we performed sex-specific analysis of *HFE* expression effects. In this case, the Cox proportional hazard model was adjusted for diagnosis age, sex, and MGMT methylation status.

### Cell Culture

Syngeneic mouse GBM cell lines (CT2A, GL261, and KR158) were grown in adherent conditions in RPMI 1640 media with 10% fetal bovine serum and 1% penicillin–streptomycin. Media was replaced every other day, and cells were passaged with Accutase and phosphate-buffered saline when sub-confluent (70%–80%). Cells were maintained in humidified incubators at 37°C and 5% CO_2_. Mouse astrocytes were derived from 3-day-old C57BL/6 neonatal mice as previously demonstrated.^26^ Human cell line 3832 was grown in suspension in supplemented neurobasal medium (neurobasal medium [Life Technologies] with 2% B27 [Life Technologies], 1% penicillin/streptomycin [Life Technologies], 1 mM sodium pyruvate [Life Technologies], 2 mM l-glutamine, 20 ng/mL EGF [R&D Systems], and 20 ng/mL FGF-2 [R&D Systems]).

### Cell Treatment With DFO and FAC

Deferoxamine (DFO; Sigma; D9533) was reconstituted in DMSO at a concentration of 76 mM and further diluted to a 5 mM stock solution in water. Ferric ammonium citrate (FAC; Sigma; RES20400-A7) was diluted in water at a concentration of 5 mM. Cells were treated with 10 μM DFO or 15 μM FAC for 3 days prior to collection and counting.

### Cell Viability

For trypan blue exclusion, cells were washed with PBS, trypsinized, collected, and spun down at 300×*g* for 5 min. Cells were then resuspended in media, and 20 μL of cells were taken for counting. An equal volume of trypan blue (20 μL) was added to the cells and mixed thoroughly. A total of 10 μL of the mixture was applied to a cell counting slide (Bio-Rad; 1450003) and measured using a Bio-Rad Automated Cell Counter.

### Radioactive Iron Uptake

^55^Fe uptake was performed as previously described.^27^ Cells were grown to 70%–80% confluence, washed, and incubated in serum-free RPMI 1640 medium for 24 h. The cells were incubated with ^55^Fe-NTA (Perkin Elmer; NEZ043001MC) in the same medium for 4 h at 37°C in a 5% CO_2_ incubator. The medium was aspirated and the cells were washed twice with 150 μM NaCl 100 μm EDTA to remove excess iron. ^55^Fe-NTA uptake was measured in triplicate wells by lysis in RIPA buffer followed by liquid scintillation counting. All values were normalized to total protein concentration as determined by Bradford assay.

### Quantitative Real-Time PCR

RNA was extracted from cells using an RNeasy kit (Qiagen; 74134), and concentrations were measured using a NanoDrop spectrophotometer. cDNA was synthesized using qScript cDNA SuperMix (Quanta Biosciences; 101414-102). qPCR was performed in Fast SYBR Green Mastermix (Applied Biosystems; 01120793) and an Applied Biosystems QuantStudio 3. Primer sequences are shown in Supplementary Table 3. During qPCR analysis, threshold cycle values were normalized to *Gapdh*.

### Hfe Knockdown and Overexpression

MISSION pLKO.1-puro Non-Mammalian shRNA Control Plasmid (SHC002) and *Hfe* shRNA plasmids TRCN0000105417 (KD1) and TRCN0000105419 (KD2) were purchased from Sigma. Lentivirus was packaged in 293T cells with psPAX2 and pMD2.G using calcium phosphate transfection. Lentiviral particles were collected from media and concentrated using a PEGit virus precipitation solution. For viral transduction, cells were grown in 10-cm tissue culture plates, and concentrated lentivirus was added to cells with the addition of 1:100 concentration of polybrene (Sigma; TR-1003). After 24 h of incubation, media was changed, and cells were incubated for an additional 24 h prior to selection with puromycin (5 μg/mL).

For human cell model 3832, *HFE* shRNA plasmids TRCN000060018, TRCN000060019, TRCN000060020, TRCN000060021, and TRCN000060022 were purchased from Sigma. Lentivirus was packaged in 293T cells with psPAX2 and pMD2.G using calcium phosphate transfection. Lentiviral particles were collected from media and concentrated using a PEGit virus precipitation solution. For transfection, cells were grown in 6-well tissue culture plates, and concentrated lentivirus was added to cells. After 24 h of incubation, media was changed and cells were incubated for an additional 24 h prior to selection with puromycin (2 μg/mL). Control MISSION pLKO.1-puro Non-Mammalian shRNA Control Plasmid (SHC002) was also used.

*Hfe* overexpression virus Lentifect Purified Lentiviral Particles were purchased from Genecopoeia (17-Lv-105-200) with the corresponding control (LP105-200).

### Intracranial Tumor Implantation

Intracranial implantation experiments with syngeneic tumor cell lines were performed as previously described.^28^ Six-week-old C57BL/6 male and female mice were anesthetized using inhaled isoflurane, and an insulin syringe attached to a stereotaxic apparatus was used to inject cells into the left hemisphere at a depth of approximately 3.5 mm. Each syringe was prepared with equal cell numbers suspended in 10 μL of null RPMI 1640 media (20 000 KR158 cells transfected with shcontrol or *Hfe* KD2; 10 000 CT2A or GL261 cells transfected with control vector or *Hfe* overexpression). Animals were monitored over time for the presentation of neurological and behavioral symptoms associated with endpoint. Investigators were blinded to experimental conditions while monitoring animals. All animal experiments were performed in compliance with institutional guidelines and were approved by the Institutional Animal Care and Use Committee of the Cleveland Clinic (protocol 2019-2195).

### Immunophenotyping by Flow Cytometry

At the indicated time points, immune cell profiling was performed on the mice bearing *Hfe* overexpressing or knockdown glioma cells when endpoint symptoms were present. Animals were euthanized by CO_2_ asphyxiation, and tumor-bearing brain, blood, and bone marrow were collected. Single-cell suspensions were prepared from the tumor-bearing hemisphere by enzymatic digestion using collagenase IV (Sigma) and DNase I (Sigma), followed by filtering through a 40-µm cell strainer. Blood was collected in EDTA-containing capillary tubes (RAM Scientific), followed by RBC lysis (BioLegend). Bone marrow was flushed from femur and tibia and filtered through a 40-µm cell strainer. For flow cytometry analysis, cells were stained with LIVE/DEAD stain (Thermo Fisher) on ice for 15 min in dark. Following washing step with PBS, surface staining was performed on ice for 30 min in the dark with the following anti-mouse monoclonal antibodies diluted in Brilliant Buffer (BD Biosciences): CD45, CD11b, CD11c, B220, NK1.1, CD3, CD4, CD8, Ly6G, Ly6C, I-A/I-E, CD69, PD-1, and LAG3. After incubation, cells were washed and fixed with FOXP3 Fix/Permeabilization buffer (eBioscience) at 4°C overnight. Intracellular staining was performed with the following antibodies diluted in FOXP3 permeabilization buffer: Foxp3, Ki-67, CD206, CD68, CTLA-4, IFN-γ, TNF, and Granzyme B. For cytokine expression, cells were stimulated with Cell Stimulation Cocktail containing protein transport inhibitor (eBiosicence) for 4 h at 37°C and subjected to staining as described above. Stained cells were acquired by a spectral cytometer Aurora (Cytek), and the acquired data were analyzed with FlowJo software (v10, BD Biosciences).

### Statistical Analysis

For 2-group comparisons, *P* values were calculated using unpaired *t* test. For multiple comparisons within one condition, a one-way ANOVA with Dunnett’s multiple comparisons test was used. For multiple comparisons in multiple conditions, a 2-way ANOVA with Tukey’s multiple comparisons test was used. Log-rank tests were used for in vivo survival analysis. All statistical analyses were performed using GraphPad Prism 9. All in vitro experiments were carried out in at least technical triplicate for each experimental group. Additional statistical details, including *P* values and sample size, can be found in figure legends.

## Results

### HFE is Upregulated in GBM Tumors and is Associated With Worse Survival in Female Patients

**Figure 1.**
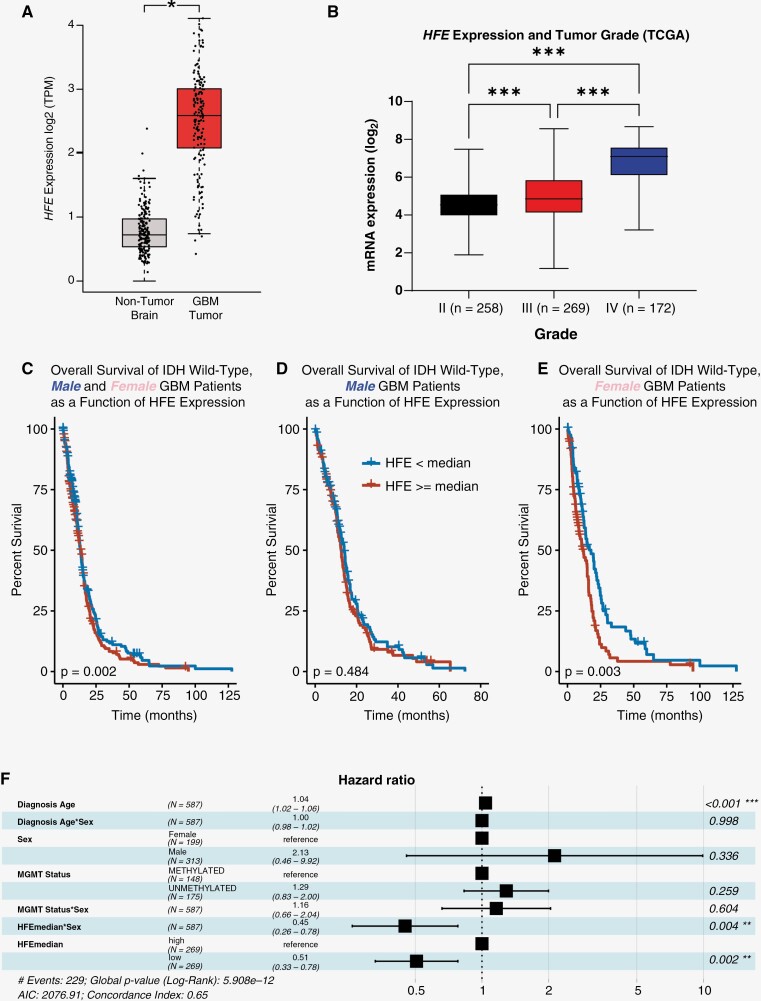
Homeostatic iron regulator (HFE) is expressed in glioblastoma (GBM) and correlates with poorer survival in female patients. (A) *HFE* mRNA expression from GEPIA in nontumor brain tissue (*left, n* = 207) compared to GBM tumor tissue (*right, n* = 163). (B) *HFE* mRNA expression from TCGA compared among glioma grades II–IV. (C) Overall survival data from *IDH1* wild-type GBM patients stratified by high vs low *HFE* mRNA expression from TCGA microarray data. Male (D) and female (E) survival data from the same data set stratified by high vs low *HFE* mRNA expression. (F) Forest plot indicates that *HFE* expression is correlated with age and sex.

Homeostatic iron regulator is a critical iron regulator, and its expression has been reported to correlate with GBM patient survival, although no mechanistic description has yet emerged to explain these observations.^22^ We therefore investigated the role of HFE in GBM by first determining whether *HFE* levels in GBM patient tumors differ from those in nontumor brain tissue. We utilized the GEPIA database^29^ and found significantly elevated *HFE* expression in GBM compared to nontumor brain tissue (Figure 1A). Expression data from The Cancer Genome Atlas (TCGA) revealed a direct correlation between *HFE* and tumor grade, with the highest expression seen in GBM (grade IV) (Figure 1B). Using these data sets, we sought to investigate the survival effects of high (greater than median) and low (lower than median) *HFE* expression on overall survival in TCGA. In isocitrate dehydrogenase 1 (*IDH1*) wild-type GBM patients, we found a statistically significant difference in survival, with high-*HFE* patients displaying a poorer prognosis (Figure 1C). While no differences were observed in male GBM patients (Figure 1D), high *HFE* expression correlated with truncated survival in female patients (Figure 1E), confirming previous findings^22^ and likely the cause of the difference observed in overall survival when not stratified by biological sex. Both *HFE* expression group assignment and the interaction term of *HFE* expression group with sex significantly affected observed survival of *IDH1* wild-type GBM patient groups. Further, we confirmed that this effect was sex-specific (Figure 1F, Supplementary Table 1) and not driven by MGMT methylation status (Supplementary Figure 1).

As HFE primarily regulates iron status through modulation of other proteins, we also analyzed survival based on high and low expression of these iron-associated genes (Supplementary Table 2). Ferritin is an evolutionarily conserved iron storage protein. Similar associations between ferritin expression and survival in GBM have previously been reported, with a reduction of ferritin expression leading to reduced tumor cell growth.^16^ Importantly, we found that high expression of the ferritin subunits ferritin heavy chain (*FTH*) and ferritin light chain (*FTL*) predicted shorter survival in female GBM patients, while no differences were present in male patients. These data suggest an important tumor-intrinsic role for HFE in GBM that may contribute to sex-specific effects on survival.

### Hfe Knockdown in Mouse Glioma Cells Induces Apoptosis in Vitro and Inhibits Tumor Initiation in Vivo

**Figure 2.**
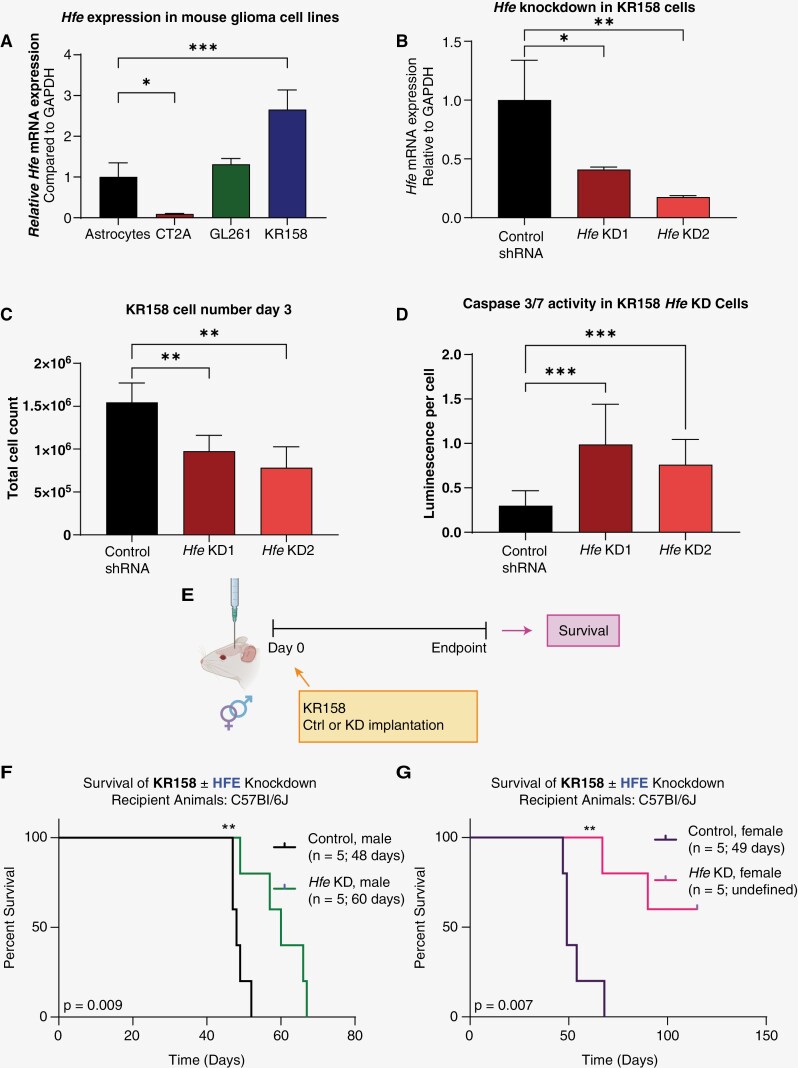
*Hfe* knockdown in KR158 cells induces apoptosis and extends survival in preclinical models. (A) RT-qPCR quantification of *Hfe* in mouse glioma cell models (CT2A, GL261, and KR158) and wild-type mouse astrocytes, normalized to *Gapdh*. (B) RT-qPCR validation of *Hfe* knockdown in KR158 cells with *Gapdh* as control. (C) Trypan blue exclusion was used to determine cell number in KR158 control and knockdown cells after 3 days of growth. (D) Caspase 3/7 activity was measured after 3 days using Caspase-Glo and normalized to cell number. (E) Schematic of KR158-knockdown experiment (created with BioRender.com). Kaplan-Meier survival curves of KR158-knockdown and control cells intracranially implanted in male (F) and female (G) mice; *n* = 5 for each group; median survival is provided on plots. *P < .05; **P < .01; ***P < .001 determined by one-way ANOVA with Dunnett’s multiple comparisons test or log-rank test for survival data (F, G). Error bars represent standard deviation.

While *HFE* expression has been reported to inform GBM survival,^22^ HFE function in tumor cells remains unclear. To directly assess HFE function, we genetically manipulated *Hfe* levels in tumor cells to determine the effect on cell growth and survival. We first evaluated baseline *Hfe* mRNA levels in 3 syngeneic mouse GBM models (CT2A, GL261, and KR158) compared to wild-type mouse astrocytes (Figure 2A). CT2A *Hfe* expression was significantly lower than that of astrocytes, while KR158 had significantly higher expression compared to mouse astrocytes. Based on these results, we utilized 2 separate, nonoverlapping shRNA constructs to perform genetic knockdown of *Hfe* in KR158 cells. Knockdown 1 (KD1) and knockdown 2 (KD2) reduced *Hfe* mRNA expression by approximately 60% and 80%, respectively, compared to a nontargeting shRNA (Figure 2B). This result was also confirmed in human GBM model 3832, where cell viability was also decreased with *HFE* knockdown (Supplementary Figure 3C and D). To determine the impact of *Hfe* knockdown on tumor cell growth in vitro, we first performed a trypan blue-exclusion assay and cell counts. By day 3 of growth, significantly fewer cells were present in both *Hfe* knockdown groups compared to the control (Figure 2C), and caspase 3/7 activity, a surrogate of apoptosis, was significantly increased in both knockdown cell lines, suggesting greater induction of cell death with *Hfe* reduction (Figure 2D). To directly quantify proliferation, we performed a dye-dilution assay in which cells were incubated with carboxyfluorescein succinimidyl ester (CFSE), a fluorescent dye that stains cells, and grown in culture. The cells were then collected, and fluorescence was quantified. Rapidly proliferating cells have a lower concentration of dye as it becomes diluted with each subsequent division, while slowly dividing cells have a higher dye concentration (Supplementary Figure 2A). CFSE dilution was significantly elevated with *Hfe* knockdown, indicating that the decreased cell number may also be driven by a decrease in the rate of proliferation (Supplementary Figure 2B). Based on the observation that elevated *HFE* expression in female GBM patients is predictive of poorer survival, we intracranially implanted control and *Hfe*-knockdown cells into male and female C57BL/6 mice to determine whether *Hfe* loss alters tumor initiation (Figure 2E). We found increased tumor latency with *Hfe* knockdown in both male and female recipient mice (Figure 2F and G). Interestingly, 3 of the female mice implanted with KD2 cells failed to present with symptoms after 100 days, at which point exploratory necropsy failed to reveal the presence of a tumor (*data not shown*). This trend of extended median survival was maintained when *Hfe* was knocked down in CT2A, which expresses a lower level of *Hfe* compared to KR158, with a longer tumor latency in KD2 conditions, which was the stronger knockdown (Supplementary Figure 2C and D). Taken together, these data suggest that loss of *Hfe* induces an apoptotic phenotype in mouse glioma cells and reduces tumor initiation in vivo.

### Increased Hfe Expression in Mouse Glioma Cells Drives Proliferation and Tumor Initiation in Vivo

**Figure 3.**
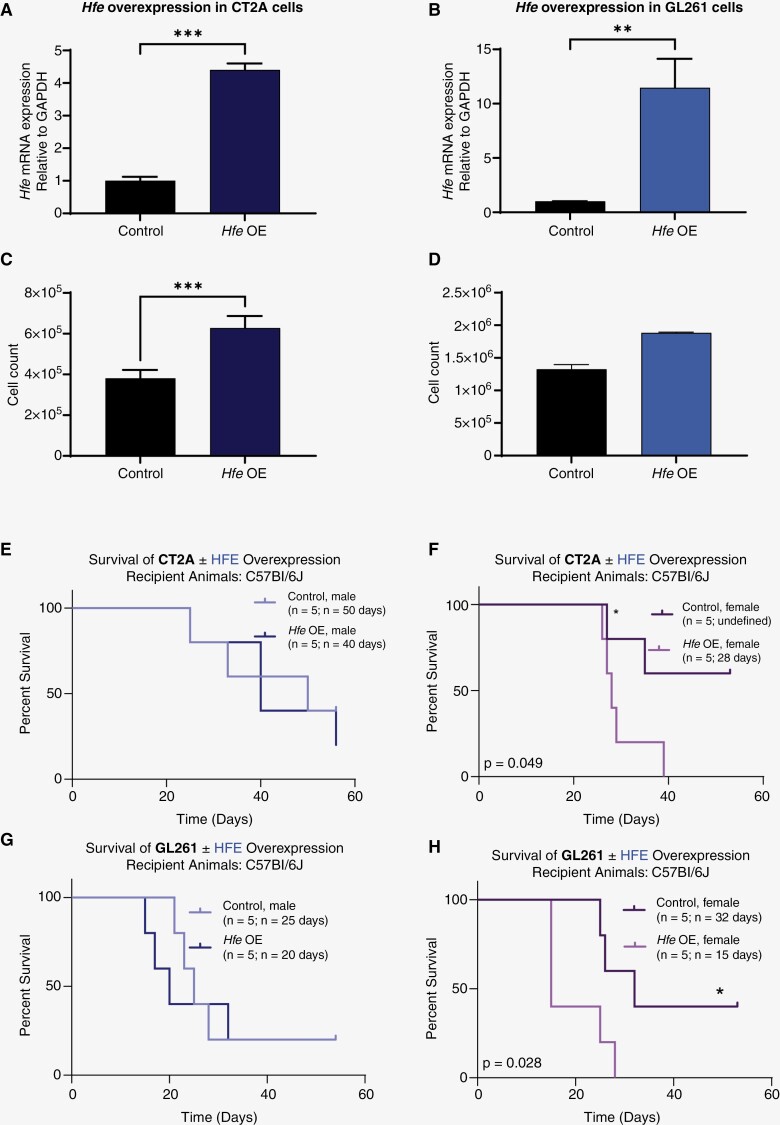
*Hfe* overexpression increases proliferation and tumor growth in preclinical models. RT-qPCR of *Hfe* overexpression in CT2A (A) and GL261 (B) models with *Gapdh* as control. Trypan blue exclusion was used to determine cell number in CT2A (C) and GL261 (D) control and *Hfe*-overexpressing cells after 5 days of growth. Kaplan-Meier survival curves of CT-2A (E, G) and GL261 (F, H) control and *Hfe*-overexpressing cells intracranially implanted into male (F, G) and female (E, G) mice. *n* = 5 for each group; median survival is provided on plots. *P < .05; **P < .01; ***P < .001 determined by *t* test (A–D) or log-rank test (E–H) for survival data. Error bars represent standard deviation.

Based on the observation that CT2A and GL261 cells express lower and comparable levels of *Hfe*, respectively, compared to astrocytes, we performed genetic overexpression of *Hfe* in these cells using stable lentiviral transduction. Overexpression was validated by RT-qPCR in both models (Figure 3A and B). Increased cell number as assessed by trypan blue exclusion was observed at day 5 (Figure 3C and D). To determine whether enhanced proliferation driven by *Hfe* overexpression could account for the differences in cell number, we assayed cell division by measuring CFSE dye dilution. *Hfe* overexpression in both CT2A and GL261 cells significantly increased the proliferation rate compared to controls (Supplementary Figure 3A), with no difference in caspase activity (Supplementary Figure 3B). To determine the impact of this perturbation on in vivo tumor initiation, male and female mice were transplanted with vector control or *Hfe* overexpression cells from both CT2A and GL261 models. Female mice implanted with both overexpression cell lines succumbed to disease more quickly than those implanted with control cells, while males exhibited no difference in survival outcomes with overexpression, suggesting that tumor growth is augmented only in females when *Hfe* levels are high (Figure 3E–H). These findings are consistent with the results observed in *Hfe*-knockdown conditions and reveal a role for Hfe in tumor cell proliferation and tumor initiation capacity in females.

### HFE Overexpression Impacts the Immune Activation State in a Sex-Specific Manner

**Figure 4.**
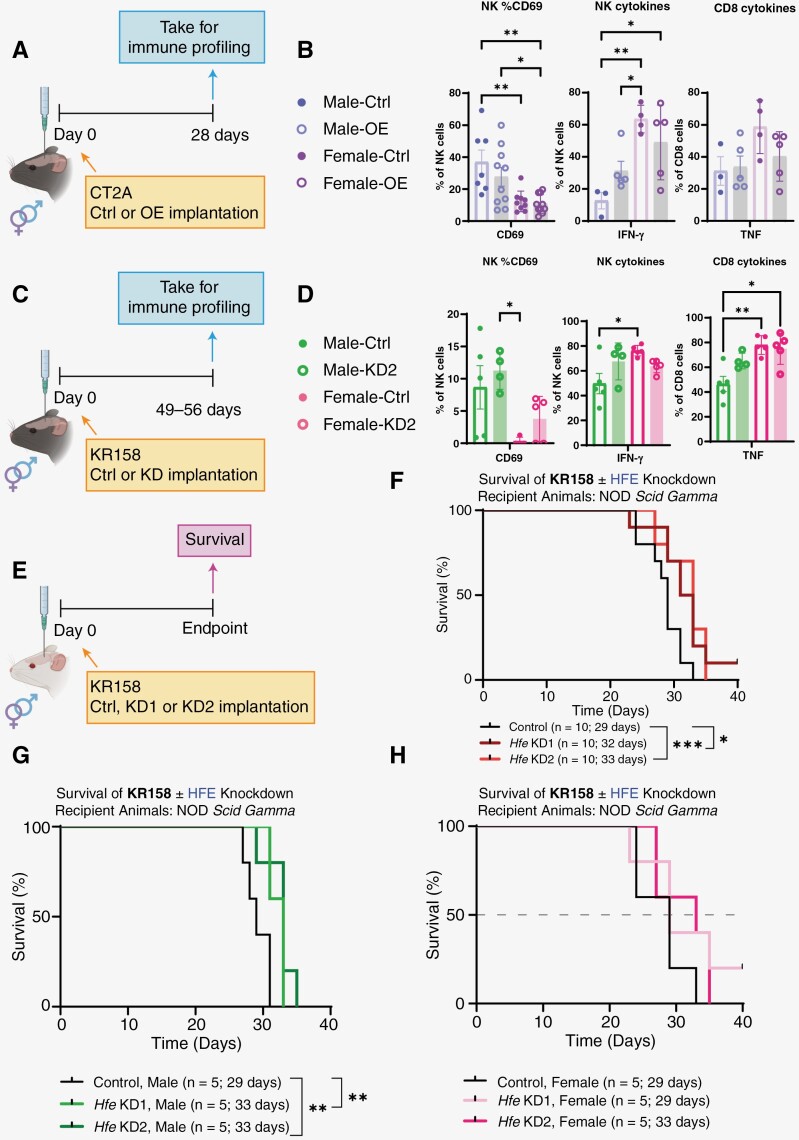
Sex-specific *Hfe* alterations in the tumor microenvironment. (A) Schematic of C57BL/6 mice implanted with CT2A control or *Hfe*-overexpressing cells. Tumor-bearing left hemispheres were taken on day 28 for immune profiling and analyzed via flow cytometry. (B) Frequencies of CD69 from NK cells, IFN-y production from NK cytokines, and TNF production from CD8+ T cells. (C) Schematic of C57BL/6 mice implanted with KR158 control or *Hfe*-knockdown cells. Tumor-bearing left hemispheres were taken on day 49 for males or day 56 for females for immune profiling and analyzed via flow cytometry. (D) Frequencies of CD69 from NK cells, IFN-y production from NK cytokines, and TNF production from CD8+ T cells. (E) Schematic of NSG mice implanted with KR158 control or *Hfe*-knockdown cells. (F) Kaplan-Meier survival curves of KR158 control and *Hfe*-knockdown cells intracranially implanted in male (G) and female (H) mice. *n* = 5 for each group; median survival is provided on plots. *P < .05; **P < .01; ***P < .001 one-way ANOVA with Dunnett’s multiple comparisons test or log-rank test for survival data. Error bars represent standard deviation.

To better understand how *Hfe* alters survival in the context of sex differences, we performed immune profiling on mice implanted with both *Hfe* knockdown and *Hfe* overexpression constructs at an intermediate timepoint prior to the development of neurological symptoms, representing the experimental endpoint. We implanted cells with *Hfe* overexpression in C57BL/6 mice and performed immune-cell profiling on day 28 posttumor implantation (Figure 4A). We did not observe any clear differences in the frequency of infiltrated immune cells between male and females in these conditions (Supplementary Figure 4G–K). However, higher frequencies of CD69, an activation and cytotoxic functional marker, were observed in the natural killer (NK) cell population in males compared to females. Conversely, female NK cells expressed higher IFN-y compared to male NK cells. There was no difference in TNF production in CD8 T cells (Figure 4B). While some sex differences in cytokine expression were observed in NK cells, there was no clear difference between the control and *Hfe* overexpression group.

Next, we analyzed the immune landscape of C57BL/6 mice implanted with *Hfe-*knockdown cells to understand the contribution of immune components in the extended survival observed in Figure 2F and G. Immune profiling was performed at 49 days for males and 56 days for females when endpoint symptoms were present (Figure 4C). Similar to our observed *Hfe* overexpression results, CD69 frequency in NK cells was higher in male cells than female, but female *Hfe*-knockdown animals had increased CD69 frequency compared to female control animals (Figure 4D). IFN-γ production in NK cells was higher in control females compared to control males, and we observed a trend of increased IFN-γ production in males but not females implanted with *Hfe*-knockdown cells. Similar trends were observed in TNF expression in CD8+ T cells, while the sex differences in TNF production in baseline male and female control animals were maintained as previously observed.^7^

We then implanted *Hfe* knockdown cells in immunodeficient NOD *scid* gamma (NSG) mice and assessed for animal survival (Figure 4E). Overall, *Hfe* knockdown slightly increased survival (Figure 4F) but not to the extent observed in immune-competent recipient mice. This effect was more pronounced in male recipient mice, where median survival increased by 4 days (Figure 4G and H). Taken together with the differences in immunocompetent models (Figure 2F and G), these results suggest that immune system differences contribute to the survival differences observed between males and females with *Hfe* manipulation.

### Hfe Knockdown Increases Iron Uptake and the Production of ROS

**Figure 5.**
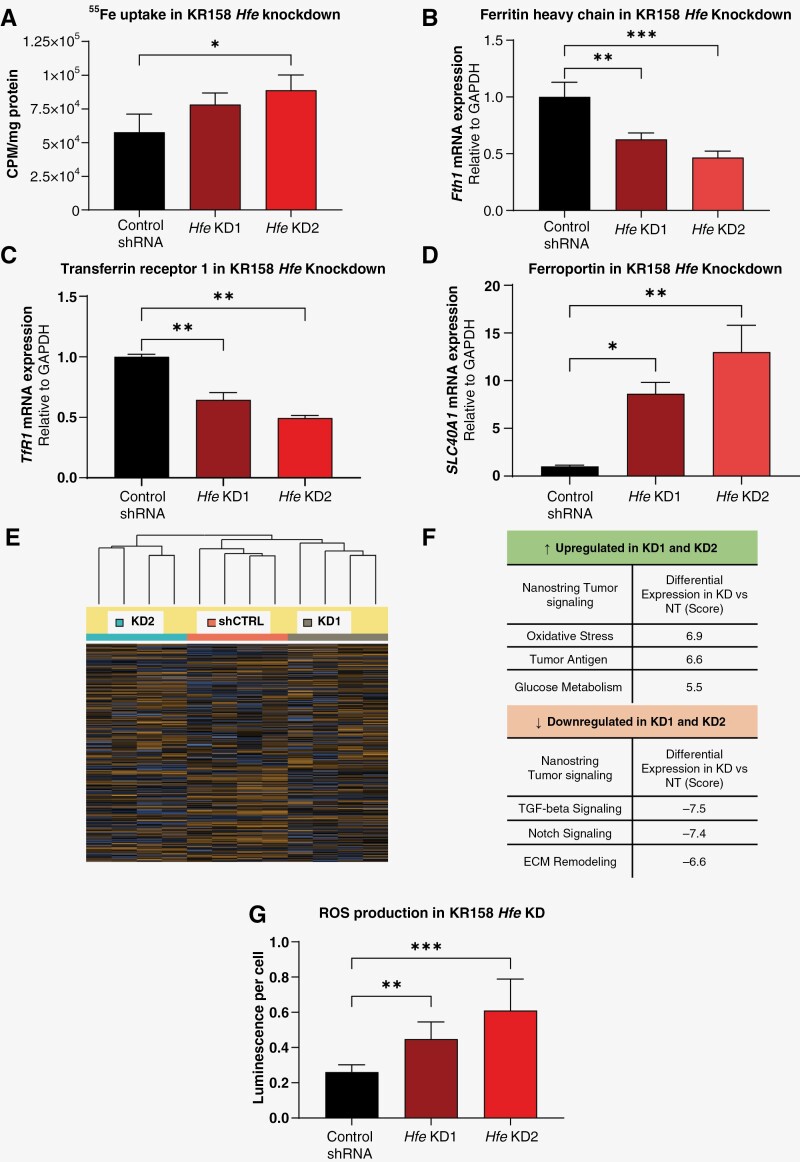
Loss of *Hfe* enhances iron uptake and induces the formation of reactive oxygen species (ROS). (A) Radioactive ^55^Fe uptake normalized to total protein content. *Hfe* knockdown construct 2 increases ^55^Fe uptake compared to control cells. (B–D) Iron-associated gene expression measured by RT-qPCR. Fold change compared to control and normalized to *Gapdh* is shown. (E) NanoString overall clustering and pathway score comparing *Hfe* knockdown to control in KR158 cells. (F) Pathway analysis demonstrates that oxidative stress is one of the more highly upregulated pathways. (G) H_2_O_2_ production in *Hfe*-knockdown and control cells was measured by ROS-Glo and normalized to cell number. *P < .05; **P < .01; ***P < .001 determined by one-way ANOVA with Dunnett’s multiple comparisons test. Error bars represent standard deviation.

As *Hfe* is also known as an iron sensor and metabolic iron regulator, we tested the functional effect of iron depletion and supplementation on tumor cell growth. We first confirmed that the presence of iron can be detected in human GBM tissue using Perl’s stain (Supplementary Figure 5A). We next treated mouse glioma cell models CT2A, GL261, and KR158 with the iron chelator DFO and iron donor FAC, which respectively inhibited and enhanced cell growth (Supplementary Figure 5B). We then investigated GBM iron uptake using radioactively labeled ^55^Fe, which revealed significantly increased uptake in *Hfe*-knockdown cells compared to controls (Figure 5A). This is consistent with the known function of HFE as a competitive inhibitor of iron uptake via transferrin receptor 1 (TFRC) binding on the cell surface. Overexpression of *Hfe* had no effect on iron uptake (Supplementary Figure 5C and D). As HFE does not directly interact with iron but rather with other iron-handling proteins, we hypothesized that modulating *Hfe* expression in these cells would in turn disrupt the expression of other iron-associated genes. We investigated mRNA levels and observed a significant reduction in the expression of ferritin heavy chain (*Fth1*) (Figure 5B) and transferrin receptor 1 (*Tfrc*) (Figure 5C), indicating that less iron was stored intracellularly. Conversely, we observed a significant upregulation of ferroportin (*Slc40a1*) in knockdown cells compared to control (Figure 5D), indicating that more iron was being exported out of the cell. This was also visualized when we used Perl’s stain to illustrate ferric (Fe^3+^) iron. Downregulation of transferrin receptor, which is involved in iron import, and upregulation of ferroportin, which is responsible for iron export, may represent an attempt by *Hfe*-knockdown cells to reduce high intracellular iron levels caused by increased uptake. Furthermore, a reduction in ferritin suggests a limited ability of these cells to store extra iron, which could result in cytotoxicity and induce apoptotic cell death. Overexpression of *Hfe* did not impact expression of transferrin receptor or ferritin, although ferroportin expression was significantly decreased in *Hfe* overexpression cells (Supplementary Figure 5E–J). When compared to control, *Hfe* knockdown conditions showed more iron deposition in the tumor microenvironment (Supplementary Figure 5K–P). RNA extraction from endpoint tumors did not show notable changes in iron regulation genes in *Hfe*-knockdown mice, with the exception of transferrin receptor in male mice implanted with *Hfe-*knockdown cells (Supplementary Figure 6).

To investigate the mechanisms through which *Hfe* loss induces an apoptotic phenotype, we interrogated signaling pathway alterations after *Hfe* knockdown using the NanoString sequencing platform (Figure 5E). Several pathways were differentially expressed when comparing control cells to *Hfe* KD1 and KD2 (Figure 5F). Among these, oxidative stress was reported as the top pathway upregulated in both knockdown cell lines compared to control. Given our findings that *Hfe* loss induces a cell death phenotype, we measured the generation of ROS in these cells and observed a significant induction of hydrogen peroxide (H_2_O_2_) production with *Hfe* knockdown compared to control (Figure 5G). H_2_O_2_-mediated apoptosis is well documented and can act through activation of caspases,^30,31^ as shown following *Hfe* knockdown (Figure 2D). Collectively, these findings suggest that loss of *Hfe* function results in an iron overload phenotype and increased production of ROS, leading to cell death.

## Discussion

Tumor cells co-opt iron metabolic programs to satisfy their high iron demand and support rapid proliferation. Targeting iron metabolism has emerged as a popular strategy for the development of clinical trials in a number of different cancers, including brain, prostate, colon, liver, and lung.^12^ These trials typically attempt to use chelating agents to reduce tumor iron availability, but their lack of specificity for tumor cells and promiscuity for other off-target divalent metal ions can lead to dose-limiting toxicities and diminish efficacy.^32–35^ Thus, a better understanding of the molecular mechanisms governing aberrant tumor iron metabolism is necessary to improve therapeutic development. GBM tumors exhibit increased iron uptake, and metabolic iron regulation in this disease has primarily been investigated from the perspective of proteins directly involved in iron handling.^16,36–38^ As an iron sensor, HFE is critical for the maintenance of intracellular iron homeostasis but indirectly regulates iron flux by binding and modulating expression of other iron-associated proteins. Previous work has described an association between increased *HFE* expression and truncated GBM patient survival.^22^ Accordingly, we find that *HFE* is upregulated in GBM tumors compared to nontumor brain tissue. However, the mechanisms underlying these observations have yet to be elucidated. One of the possible technical limitations of the cohort-based analysis is the usage of microarray gene expression data. RNA sequencing is considered to be a “gold standard” method, yet it was not available for the entire TCGA cohort, limiting statistical power. Importantly, consistency between microarray and RNA sequencing data in TCGA was not explicitly checked in our analysis, but downstream murine model experiments serve as a confirmation that our findings in TCGA could not be attributed to technical artifacts.

**Figure 6.**
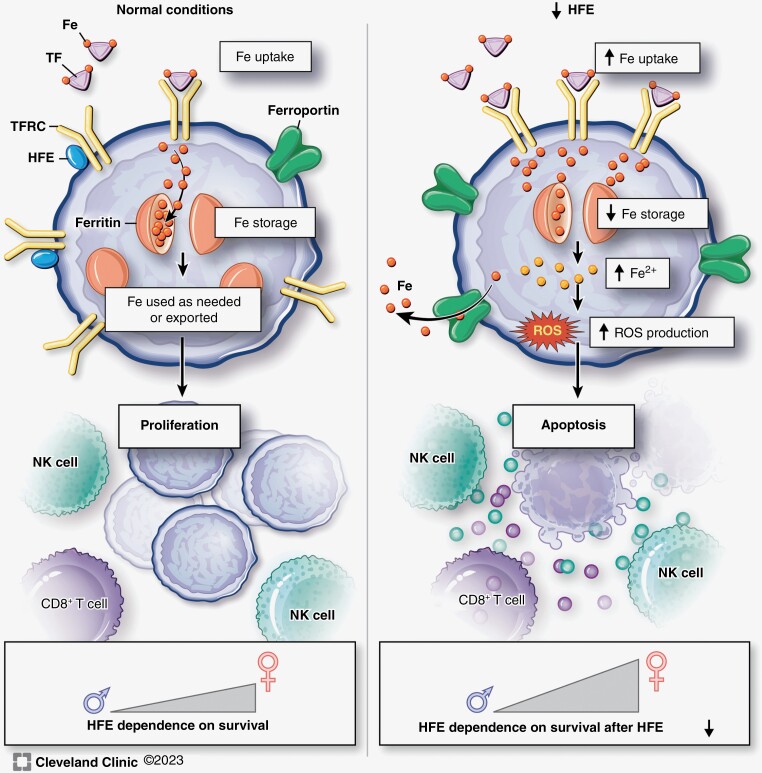
Loss of tumor cell-intrinsic *Hfe* induces cell death through reactive oxygen species (ROS) generation. Under normal conditions (left panel) homeostatic iron regulator (HFE) associates with transferrin receptor (TFRC) on the cell membrane to competitively inhibit transferrin (TF)-bound iron uptake and maintain homeostasis. Our findings indicate that loss of HFE (right panel) permits greater iron uptake and results in downregulation of ferritin, leading to reduced iron storage, greater iron in the tumor microenvironment, greater production of ROS, and increased cytokines released by NK and CD8+ T cell cells that contribute to the apoptotic phenotype. Survival becomes even more dependent on sex when *Hfe* is reduced.

Iron uptake and storage can be exploited by tumor cells to enhance iron sequestration and promote tumor growth. GBM cancer stem-like cells demonstrate increased expression of iron uptake proteins transferrin (TF) and transferrin receptor (TFRC), as well as the iron storage protein ferritin. Knocking down either subunit of ferritin (FTH1 or FTL) in these cells was sufficient to reduce tumor sphere formation in vitro and tumor initiation in vivo.^16^ We also observed that *Hfe* perturbations impact survival of animals with intracranial tumors. We did not directly test a tumor initiation versus growth dependence for *Hfe*, which could be performed with inducible *Hfe* knockdown/knockout or overexpression constructs, with assessments of in vivo iron and ROS levels, apoptosis, and expression of iron-related genes including ferritin, ferroportin, and transferrin receptor. These investigations represent the focus of future studies. Our findings demonstrate increased iron uptake with *Hfe* knockdown and a counterintuitive induction in apoptotic cell death. When we interrogated the impact of *Hfe* knockdown on iron-associated gene expression, we found a reduction in ferritin heavy chain (*Fth1*). Ferritin is essential for limiting the accumulation of intracellular ferrous iron (Fe^2+^), which can be used in the Fenton reaction to catalyze the formation of superoxide and hydroxyl radicals (Fe^2+^ + H_2_O_2_ → Fe^3+^ + HO^•^ + OH^−^).^39^ These free radicals can cause oxidative stress and induce several forms of cell death, including caspase-dependent apoptosis.^40^ Indeed, we found that *Hfe* knockdown induced ROS production in the form of H_2_O_2_, which is consistent with previous reports that ferritin degradation in human GBM cells promotes ROS generation and induces ferroptosis, an iron-dependent form of cell death.^17^ In fact, ferroptotic induction via delivery of iron oxide nanoparticles has been reported as an effective therapeutic strategy in animal models of GBM.^41^ This work demonstrated that increasing intracellular iron levels while simultaneously augmenting H_2_O_2_ production results in potent ROS generation and ferroptosis with minimal off-target toxicity.^42^ Thus, a shift in focus from iron chelation, which broadly targets iron and other metal ions, to iron storage may represent a less toxic strategy in the treatment of GBM tumors (Figure 6).

Sex is an important determinant of tumor risk and survival outcomes in GBM.^3^ Men are more likely to develop GBM at a ratio of 1.6:1 compared to females, and male GBM patients are reported to have worse prognoses, with an estimated median survival of 15.9 months compared to 22.6 months in female patients.^3^ Our work supports previous findings that tumor-intrinsic HFE exerts a sex-specific effect on overall survival, with high HFE expression ablating the survival advantage normally observed in female patients.^22^ The basis for these differences is still unclear but may be attributed to sex-mediated differences in iron metabolism. Men typically have larger iron stores than women, as much as 2- to 3-fold higher in some tissues, and women experience iron deficiency and anemia at much higher rates than men.^43,44^ Historically, this has largely been attributed to blood loss through menstruation and conditions such as pregnancy, but other factors such as hormones likely play a role. For example, there is an inverse relationship between serum ferritin and estrogen levels, which causes iron stores to gradually increase after menopause.^45^ However, both serum ferritin and iron stores are still reportedly lower in postmenopausal women than in men.^46,47^ A consequence of these sex differences in iron metabolism is that high serum iron levels are much more prevalent in men than women and have been associated with increased cancer risk.^41^ We observed truncated survival only in female mice implanted with *Hfe*-overexpressing tumor cells. This finding is consistent with human GBM clinical data and may be partially explained by sex differences in iron levels. Conversely, *Hfe* knockdown led to protracted survival in both male and female animals. We suspect that this survival benefit is mediated by the apoptotic phenotype and may therefore not discriminate based on sex despite sex differences in the immune response observed. Taken together, these results suggest a role for sex differences in HFE-mediated tumor iron regulation that ultimately results in the observed differential survival benefits. In addition, our findings indicate that *Hfe* manipulation impacts both the tumor cells in a cell-intrinsic manner as well as the immune response in a sex-specific manner, suggesting that Hfe likely serves multiple roles in GBM.

While these findings are one of the first descriptions of HFE function in GBM, this work has limitations. Due to the complex regulatory nature of HFE, it is difficult to determine the direct target and sequence of events leading to apoptosis upon *Hfe* depletion. It is possible that these effects are mediated primarily by changes in iron status with HFE modulation. However, HFE also forms cell surface protein complexes that initiate downstream signaling and may directly influence ROS production or apoptosis. Further complicating our understanding of this process is the regulation of iron-associated gene and protein expression by iron availability. The mRNA transcripts of most iron-associated genes possess iron response elements on their 5ʹ untranslated regions, which regulate translation rates. Thus, HFE may modulate expression directly, through intracellular signaling, or indirectly, through iron availability, and more insight into this complex relationship is necessary to fully appreciate its role in tumor cells. Future studies will include epistatic rescue studies in *Hfe*-manipulated conditions, including the potential requirement for ferritin heavy chain in this process, as well as the ability to rescue the effects of *Hfe* knockdown with ROS inhibition. Our studies demonstrate that *Hfe* changes impact iron regulation and many tumor cell phenotypes in vitro, but the extent to which this effect is maintained in vivo has not been extensively examined and represents a limitation of the current study. Another limitation is the influence of sex chromosomes on our mouse tumor cell lines. Upon karyotyping, we discovered that these lines have only one X chromosome, and therefore any contribution from sex chromosomes is not likely to be appreciated. Thus, our ability to thoroughly interrogate the role of tumor cell-derived sex differences is currently limited and would rely on other models in which the sex chromosome complement is intact. The development of new syngeneic mouse GBM cell lines in which the cell sex is known would be beneficial to fully appreciate these differences and represents an area for future exploration. Another limitation is that these studies rely on mouse GBM models, and this therefore limits the ability to extrapolate these findings to human GBM and human GBM cells. While we show some indication that human GBM cells are also reliant on *Hfe* for growth in vitro and there is an association with GBM patient outcome, additional mechanistic studies are required to validate these findings in human models. Despite these limitations, HFE remains an intriguing mechanism for targeting tumor iron flux. In conclusion, our findings demonstrate that HFE drives tumor cell proliferation and may be a viable approach for targeting iron metabolism and inducing cell death.

## Supplementary Material

vdad154_suppl_Supplementary_Material

vdad154_suppl_Supplementary_Data
