# Supplementary material for: Homeostatic iron regulatory protein drives glioblastoma growth via tumor cell-intrinsic and sex-specific responses
Source: Neurooncol Adv. 2023 Nov 28;6(1):vdad154. doi: 10.1093/noajnl/vdad154 (PMC10794878; doi:10.1093/noajnl/vdad154)
Supplement: vdad154_suppl_Supplementary_Material [file vdad154_suppl_supplementary_material.pdf]

Supplemental Figure 1

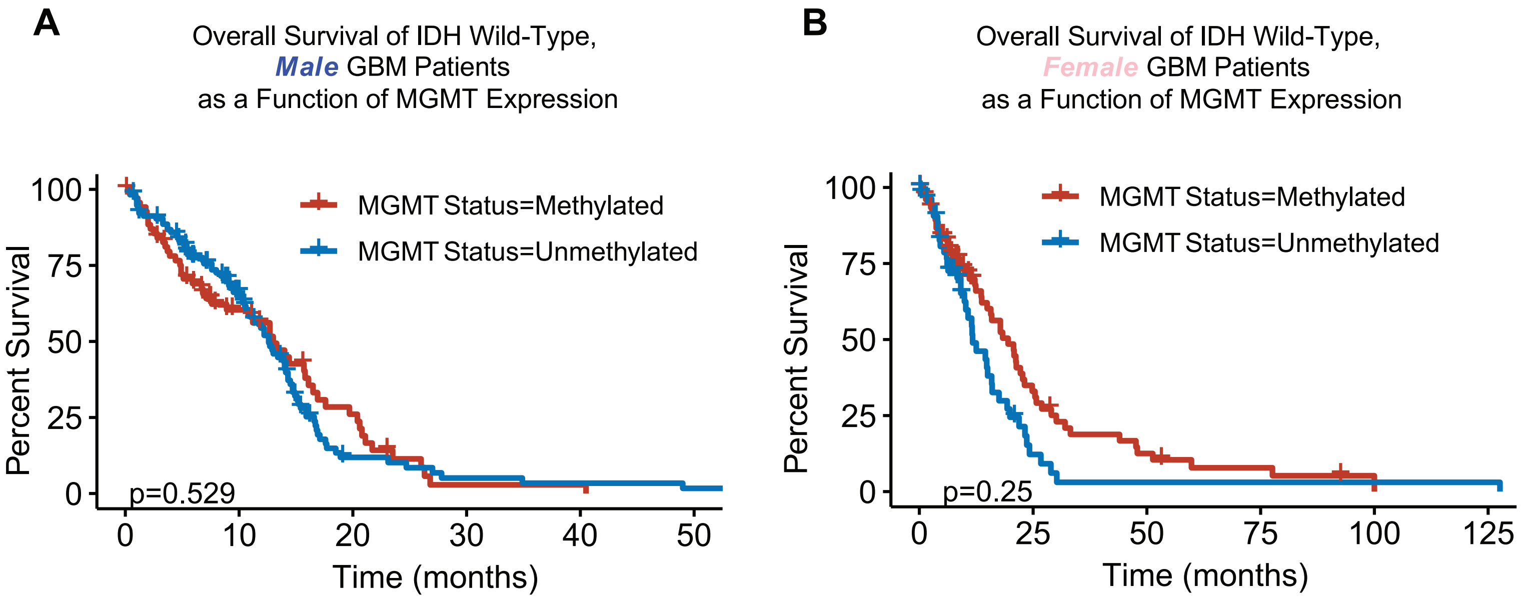

Supplemental Table 1

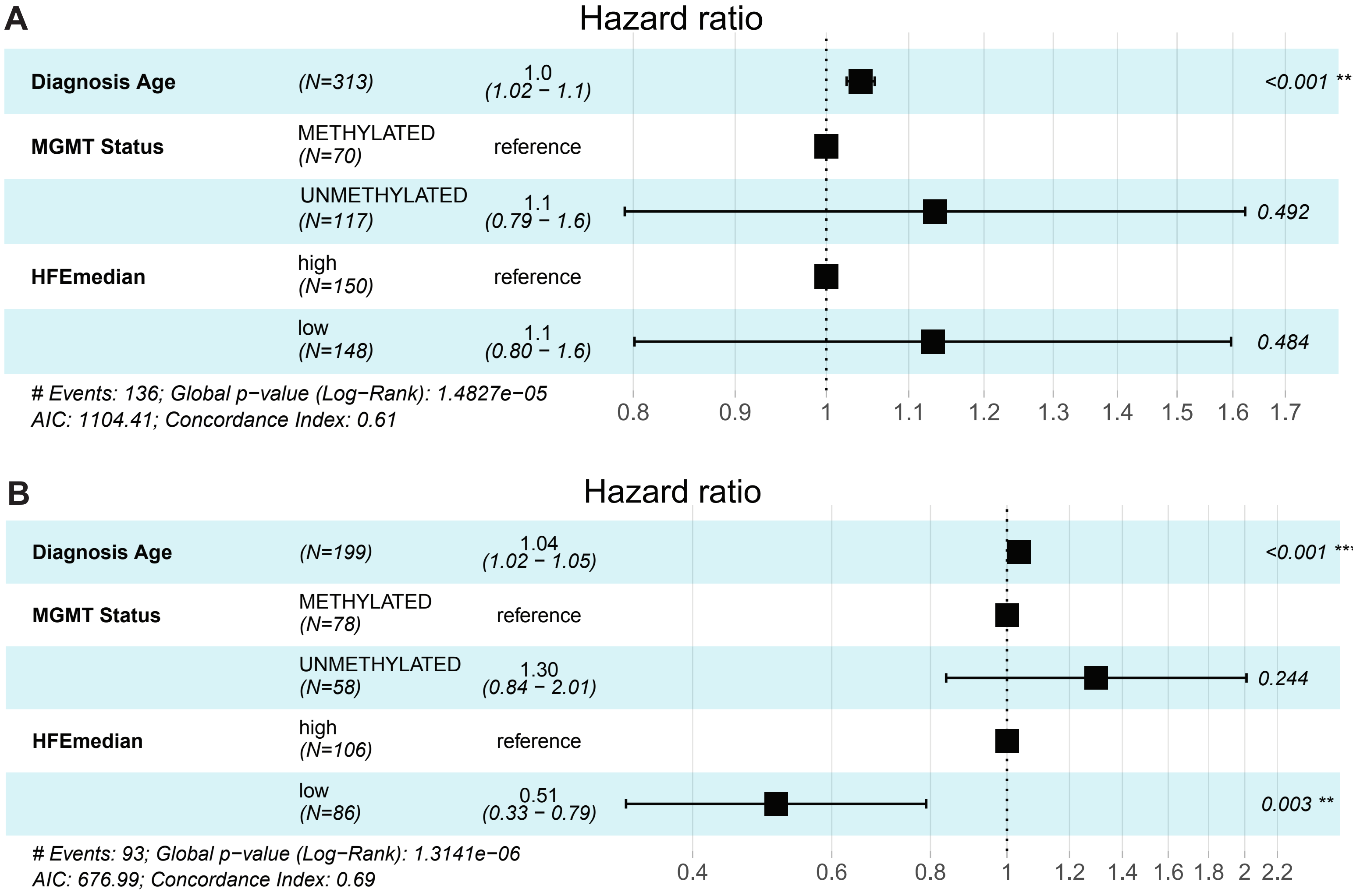

Supplemental Figure 2

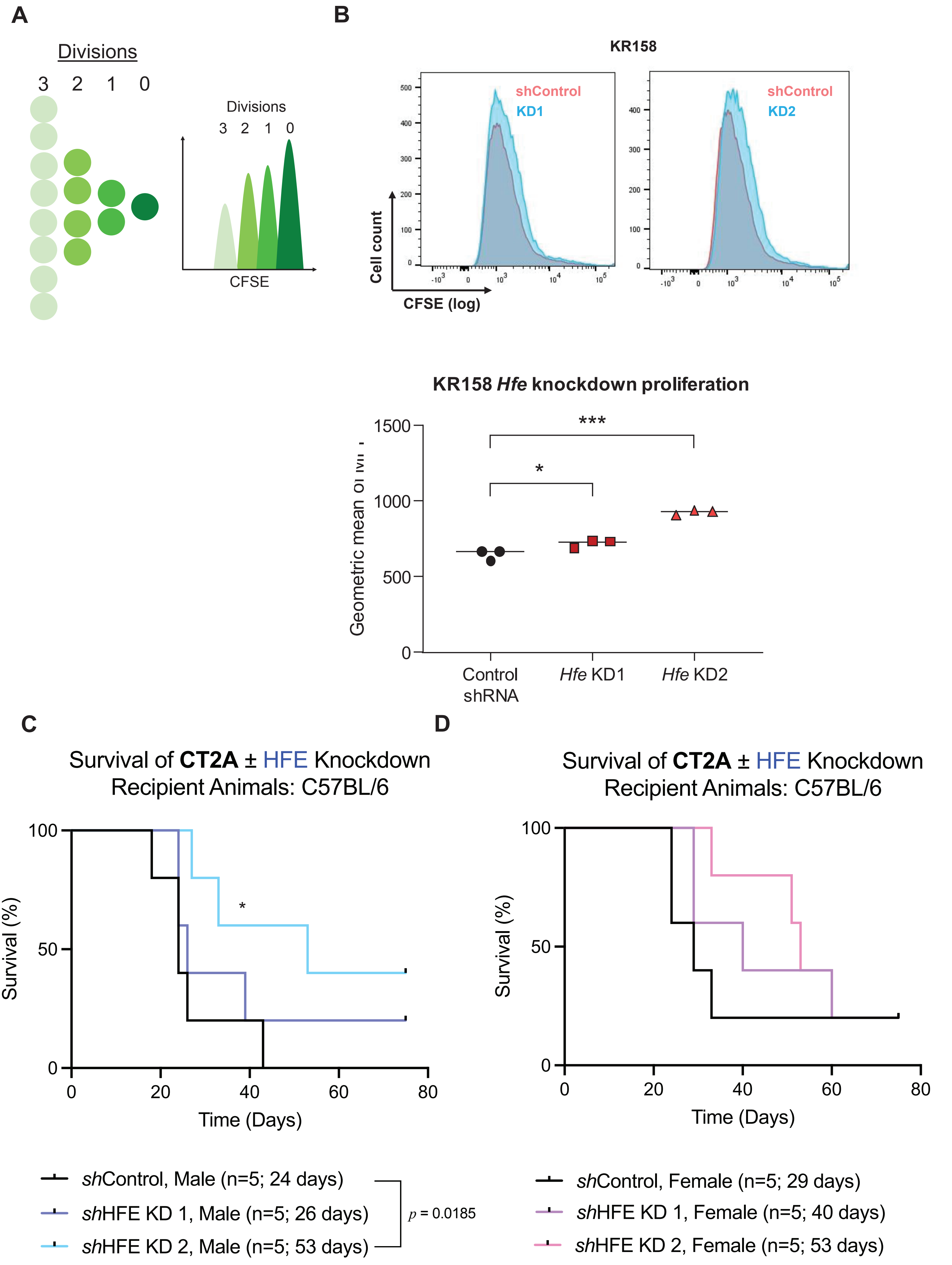

Supplemental Table 2

All patients (n=368)			
Gene	Median survival months (Low)	Median survival months (High)	P-value
FTH	14.9	12.7	**<0.05***
FTL	15.0	12.2	**<0.05***
HAMP	14.1	13.9	0.82
HFE	14.0	13.9	0.19
TFRC	13.9	13.9	0.82
Female patients (n=143)			
Gene	Median survival months (Low)	Median survival months (High)	P-value
FTH	18.3	12.6	**<0.05***
FTL	17.6	12.2	**<0.05***
HAMP	15.0	16.0	0.66
HFE	16.8	14.9	**<0.05***
TFRC	14.9	15.7	0.09
Male patients (n=225)			
Gene	Median survival months (Low)	Median survival months (High)	P-value
FTH	14.0	12.7	0.50
FTL	14.1	12.2	0.40
HAMP	13.3	12.9	0.35
HFE	13.1	12.9	0.89
TFRC	12.9	13.3	0.21

Supplemental Figure 3

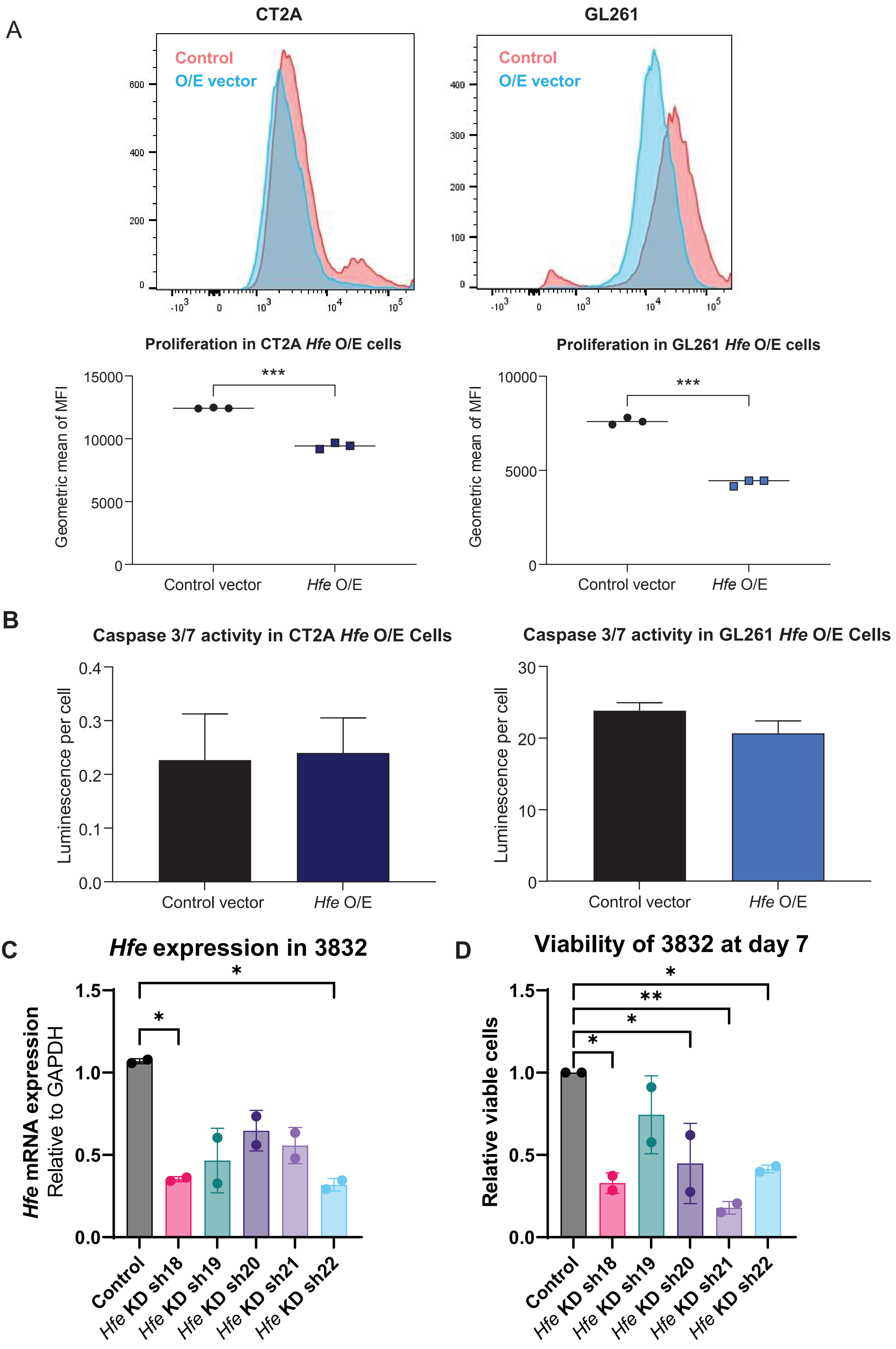

Supplemental Figure 4

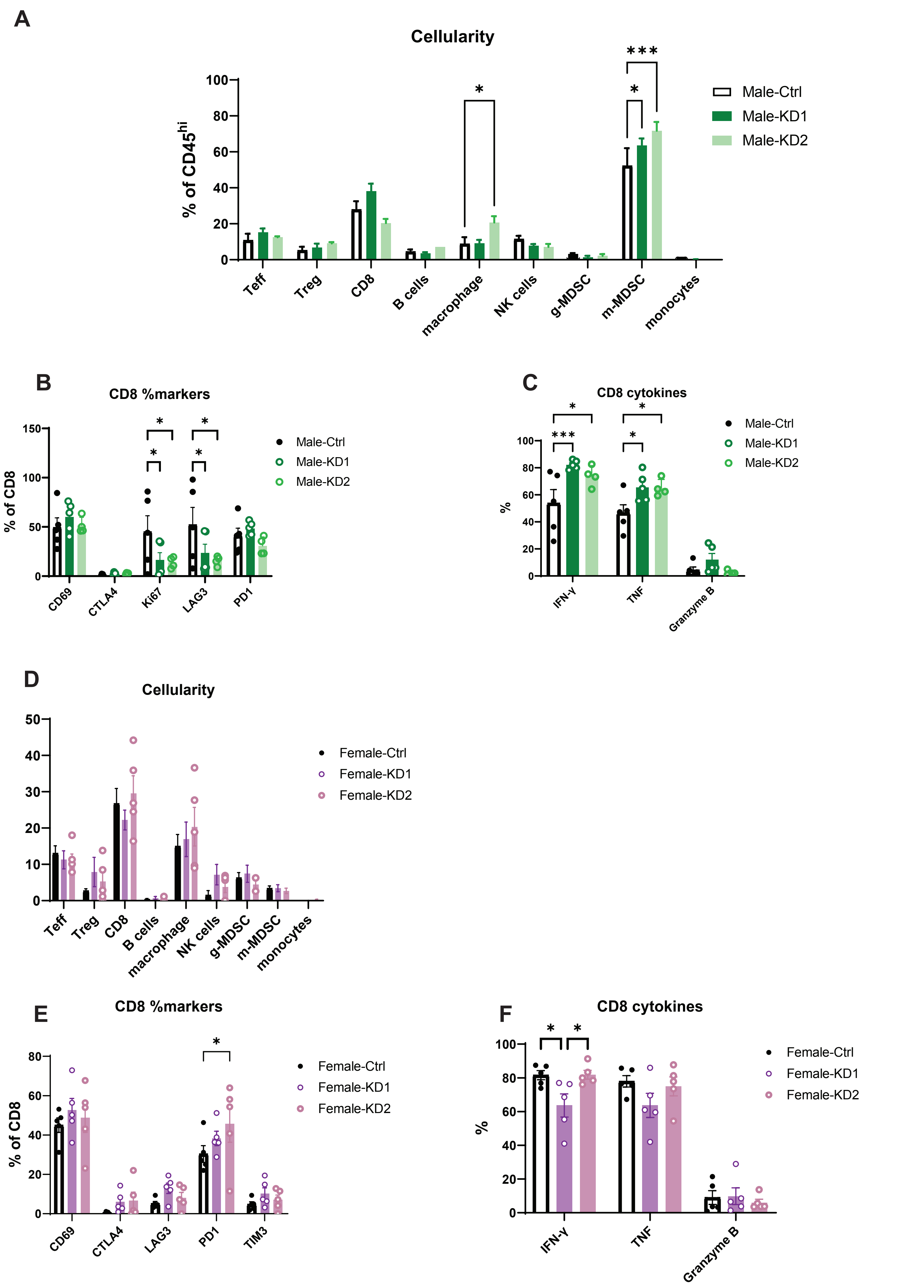

Supplemental Figure 4

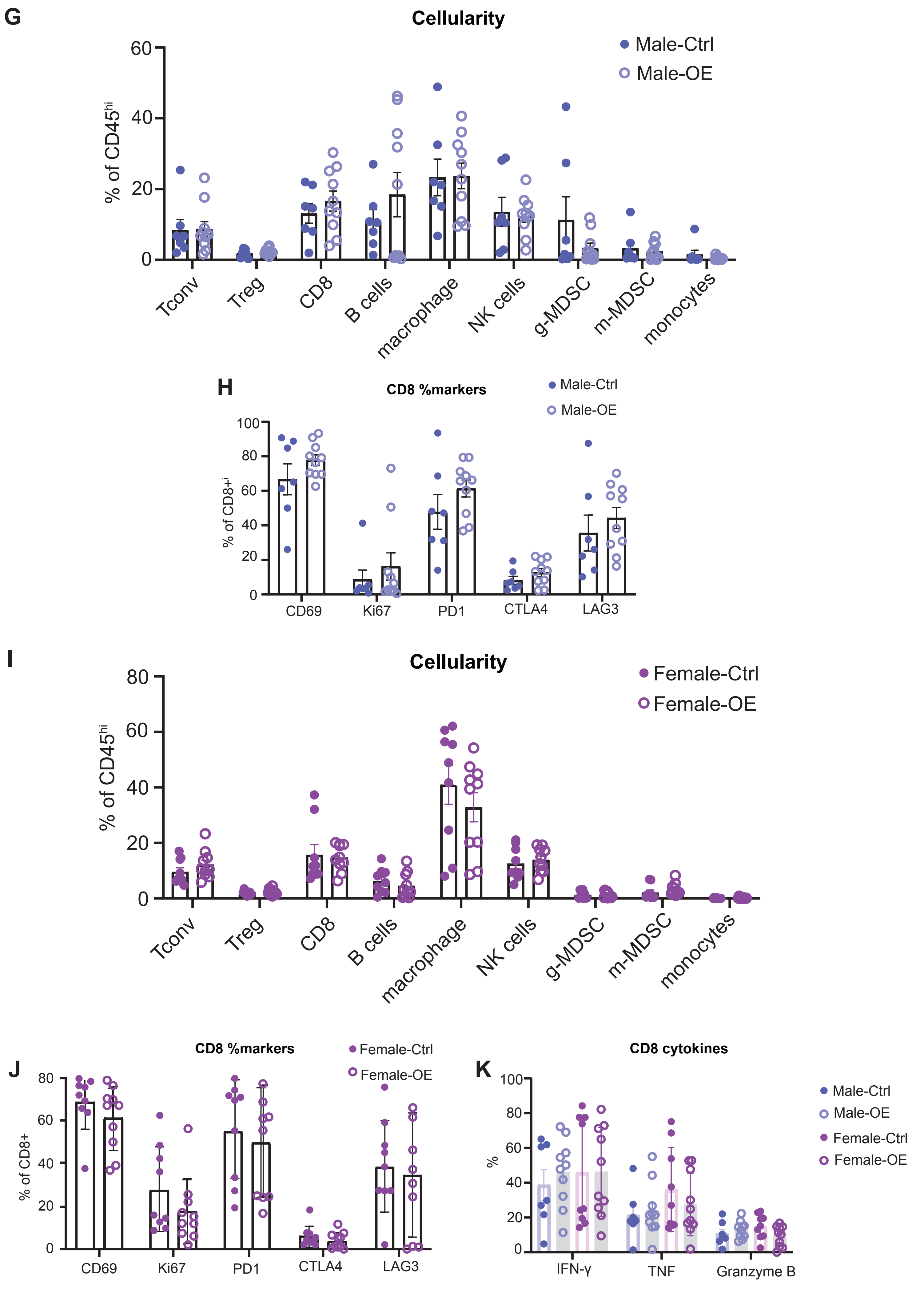

Supplemental Figure 5

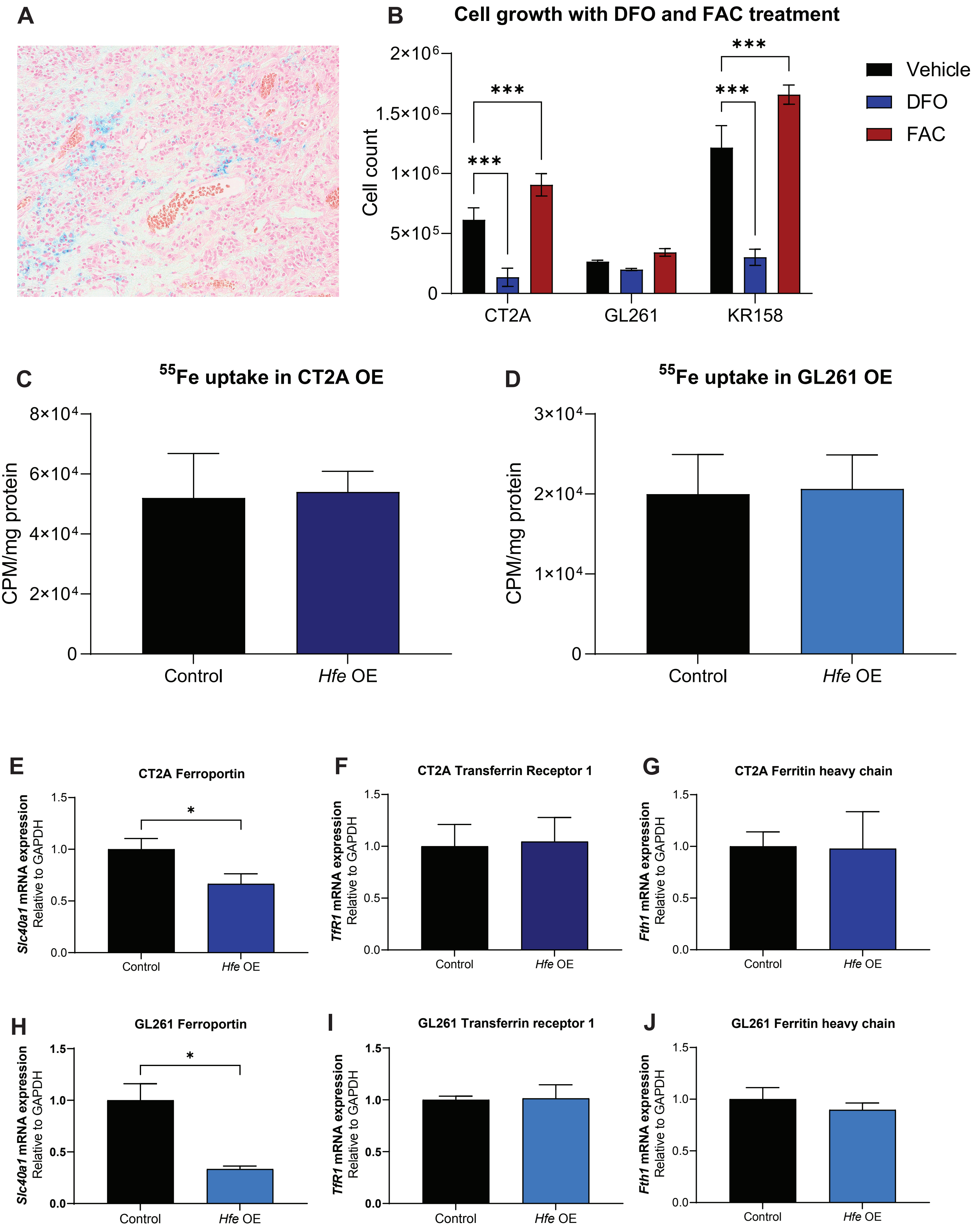

Supplemental Figure 5

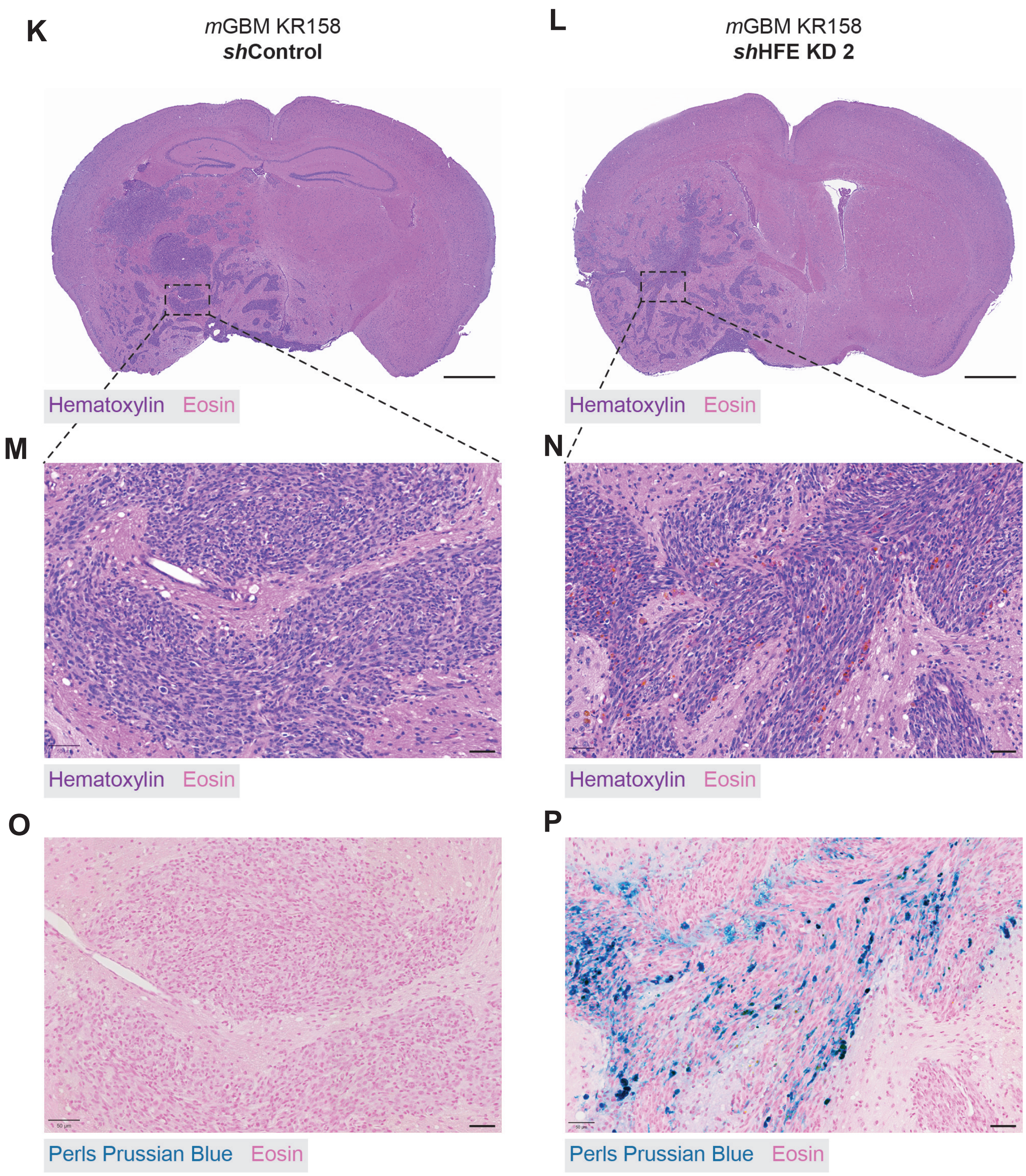

Supplemental Figure 6

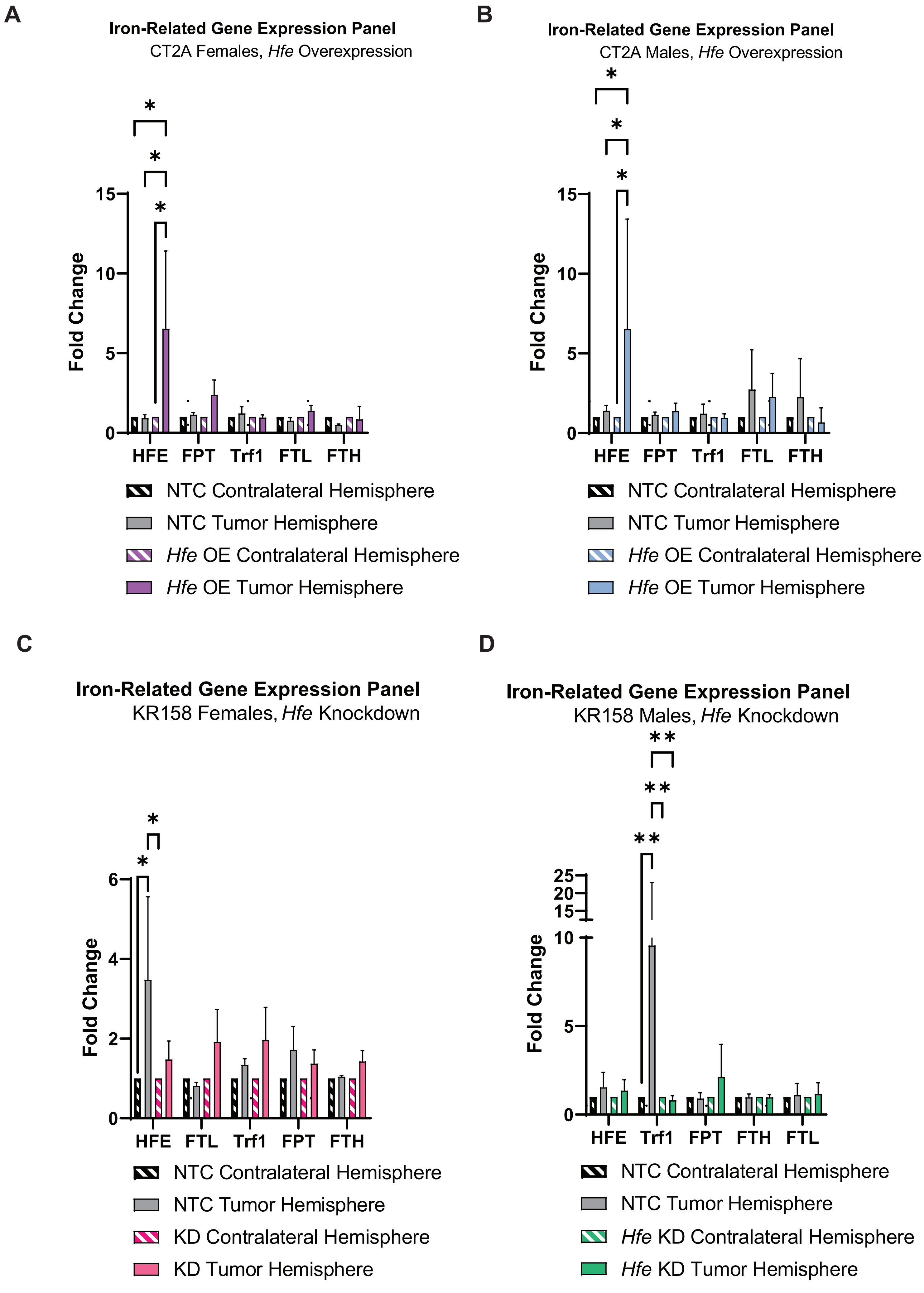

Supplemental Table 3

**Gene**	**Forward**	**Reverse**
Fth1	CTCATGAGGAGAGGGAGCAT	GTGCACACTCCATTGCATTC
Gapdh	AACAGCAACTCCCACTCT TC	CCTGTTGCTGTAGCCGTATT
Hfe	CACCGCGTTCACATTCTCTA	AAAGAGCTGGTCATCCACATAG
Slc40a1	CGGTCTTTGGTCCTTTGATTTG	GCAGAAGGTCAAGAAGGTAGTT
Tfrc	AGCCAGATCAGCATTCTCTAAC	TCTGCAGCCAGTTTCATCTC
Ftl1	CCTCGAGTTTCAGAACGATCGC	CCTGATTCAGGTTCTTCTCCATG
