# Supplementary material for: Homeostatic iron regulatory protein drives glioblastoma growth via tumor cell-intrinsic and sex-specific responses
Source: Neurooncol Adv. 2023 Nov 28;6(1):vdad154. doi: 10.1093/noajnl/vdad154 (PMC10794878; doi:10.1093/noajnl/vdad154)
Supplement: vdad154_suppl_Supplementary_Data [file vdad154_suppl_supplementary_data.pdf]

Supplemental Materials and Methods

Supplemental Figure 1.

(A) Survival of male GBM patients as a function of MGMT expression. (B) Survival of female GBM patients as a function of MGMT expression. Blue = unmethylated, red = methylated.

Supplemental Figure 2.

(A) CFSE is used to measure proliferation. During each round of cell division, intracellular CFSE becomes diluted and is quantified to determine proliferation rate. (B) Flow cytometry plots of CFSE dilution in KR158 *Hfe* knockdown cells compared to control. (C) Survival of female C57Bl/6 mice implanted with CT2A control vs CT2A *Hfe* knockdown cells. (D) Survival of male C57Bl/6 mice implanted with CT2A control vs CT2A *Hfe* knockdown cells.*p<0.05; **p<0.01; ***p<0.001 determined by t-test or log-rank test for survival data. Error bars represent standard deviation.

Supplemental Figure 3.

(A) Flow cytometry plots of CFSE dilution in CT2A and GL261 *Hfe* overexpression cells. (B) Caspase 3/7 activity measured using Caspase-Glo and normalized to cell number. (C) Hfe expression via RT-qPCR of knockdown in human cell line 3832. (D) Viability of 3832 at 7 days for control vs *Hfe* knockdown constructs via crystal violet staining.

Supplemental Figure 4.

(A) Cellularity of male C57Bl/6 mice implanted with control or *Hfe* knockdown cells. (B) CD8 markers of male C57Bl/6 mice implanted with control or *Hfe* knockdown cells. (C) CD8 cytokine expression of male C57Bl/6 mice implanted with control or *Hfe* knockdown cells. (D) Cellularity of female C57Bl/6 mice implanted with control or *Hfe* knockdown cells. (E) CD8 markers of female C57Bl/6 mice implanted with control or *Hfe* knockdown cells. (F) CD8 cytokine expression of female C57Bl/6 mice implanted with control or *Hfe* knockdown cells.

Supplemental Figure 5.

(A) Perl's Prussian blue stain to visualize iron content in human GBM tissue (scale bar at 70 µM). (B) Cell growth measured by trypan blue exclusion in mouse glioma cells (CT2A, GL261, and KR158) after treatment with vehicle (water), the chelator deferoxamine (DFO, 10 µM) or iron donating agent ferric ammonium citrate (FAC, 15 µM). 50,000 cells were plated in triplicate in 6 well plates and collected and counted at days 1, 3, 5, and 7. (C-D) Radioactive ^{55}Fe uptake normalized to total protein content in CT2A control vs *Hfe* OE cells. (E-J) Iron-associated gene expression measured by RT-qPCR. Fold change compared to GAPDH control is shown. Hematoxylin and eosin staining of male C57Bl/6 mouse implanted with (K) control cells and (L) *Hfe* knockdown cells with a tumor area highlighted in a black bounding box. (M-N) Respective staining of black bounding box in Figure 5K showing enlarged hematoxylin and eosin of area, as well as (O-P) Perl's Prussian Blue staining of the same area.

Supplemental Figure 6.

(A) Iron-related gene expression panel from RNA extraction of tumor bearing hemispheres vs non-tumor bearing hemispheres in female mice implanted with CT2A control cells or *Hfe* overexpressing cells. (B) Iron-related gene expression panel from RNA extraction of tumor bearing hemispheres vs non-tumor bearing hemispheres in C57Bl/6 male mice implanted with CT2A control cells or *Hfe* overexpressing cells. (C) Iron-related gene expression panel from RNA extraction of tumor bearing hemispheres vs non-tumor bearing hemispheres in C57Bl/6 female mice implanted with KR158 control cells or *Hfe* knockdown cells. (D) Iron-related gene expression panel from RNA extraction of tumor bearing hemispheres vs non-tumor bearing hemispheres in C57Bl/6 male mice implanted with KR158 control cells or *Hfe* knockdown cells.

Supplemental Table 1. Hazard ratio forest plots demonstrating survival effects of *Hfe* expression.

Supplemental Table 2. TCGA overall survival in male and female GBM patients with high and low expression of iron-associated genes.

Supplemental Table 3. Mouse RT-qPCR primers used in this study.

Supplemental Materials and Methods

CFSE proliferation assay

Hfe knockdown or overexpressing cells and respective controls were stained with 1 μmol/L carboxyfluorescein succinimidyl ester (CFSE; BioLegend 423801) for 15 minutes at 37°C and washed with twice with PBS. Stained cells were then plated at equal densities (50,000 KR158 and CT2A; 100,000 GL261) in 6 well plates and allowed to grow for 2 to 3 days. Samples were analyzed using a BD LSR Fortessa.

Flow cytometry

Samples were stained with LIVE/DEAD (Thermo Fisher Scientific; L34961) diluted at 1:500 in PBS for 10 minutes at room temperature. Cells were then washed once in PBS and resuspended in Mojosort Buffer (Biolegend; 480017) at a 1:5 dilution in ddH_2O. Samples were analyzed with a BD LSR Fortessa (BD Biosciences), and FlowJo (Version 10.5.0, FlowJo LLC) was used for data analysis. Compensation controls were used to account for spectral overlap.

Radioactive iron uptake

^{55}Fe uptake was performed as previously described[43]. Cells were grown to 70-80% confluence, washed, and incubated in serum-free RPMI 1640 medium for 24 h. The cells were incubated with ^{55}Fe-NTA in the same medium for 4 h at 37°C in a 5% CO_2 incubator. The medium was aspirated and the cells were washed twice with 150 μM NaCl 100 μm EDTA to remove excess iron. ^{55}Fe-NTA uptake was measured in triplicate wells by lysis

in RIPA buffer followed by liquid scintillation counting. All values were normalized to total protein concentration as determined by a Bradford assay.

Nanostring

RNA was isolated using an RNeasy mini kit (Qiagen) and the nCounter® Tumor360 Panel was subsequently used to analyze gene expression. Two non-overlapping shRNA *Hfe* constructs (KD1 and KD2) in KR15 cells were analyzed in triplicate. nSolver version 4.0 was used to normalize and analyze data to determine up- and downregulated pathways in both knockdown conditions.

ROS quantification

ROS production was quantified using the ROS-Glo H_2O_2 assay (Promega; G8820) according to the manufacturer's protocol. Cells were plated in triplicate in a 96 well plate at equal density (1,000 cells per well) and allowed to grow for 2 to 3 days prior to incubation with H_2O_2 substrate solution for 4 hours. The ROS-Glo detection solution was then added and incubated for 20 min. Luminescence was measured using a Victor3 plate reader (PerkinElmer) and values were normalized to cell number.

Cell viability assay

Cells were plated at 50,000 cells/well in a 24-well plate (ThermoFisher; 142475) at 1mL/well. Upon days 0 and 7, media was aspirated, and then cells were washed with PBS twice. 0.25% Crystal Violet with 20% methanol was added to each well, and then each well was washed twice. Upon drying, 500µL of 100% ethanol was added to each

well, sealed tightly, then rocked on an orbital shaker (ThermoFisher; 15-453-907) for one hour. After the hour, the plate was read at 570nm absorbance.

Perl's Prussian Blue staining

Staining of tumor sections with hematoxylin and eosin and Perl's Prussian blue and nuclear fast red counterstain was performed by the Lerner Research Institute Imaging Core.

Intracranial tumor implantation

Intracranial implantation experiments with syngeneic tumor cell lines were performed as previously described[42]. 6-week-old C57Bl/6J mice were anesthetized using inhaled isoflurane and an insulin syringe attached to a stereotaxic apparatus was used to inject cells into the left hemisphere at a depth of approximately 3.5mm. Each syringe was prepared with equal cell numbers suspended in 10μL of null RPMI 1640 media (20,000 KR158 cells transfected with shcontrol or *Hfe* KD2; 10,000 CT2A or GL261 cells transfected with control vector or *Hfe* overexpression). Animals were monitored over time for the presentation neurological and behavioral symptoms associated with end-point. Investigators were blinded to experimental conditions while monitoring animals. All animal experiments were performed in compliance with institutional guidelines and were approved by the Institutional Animal Care and Use Committee of the Cleveland Clinic (protocol 2019-2195).
